# A Comparison of the Outcomes Between Suprapatellar and Infrapatellar Approaches of Intramedullary Interlock Nailing in Patients With Extra-Articular Tibial Fractures

**DOI:** 10.7759/cureus.40108

**Published:** 2023-06-07

**Authors:** Nilesh Joshi, Shantanu Deshkmukh, Yash Shewale

**Affiliations:** 1 Orthopaedics and Traumatology, N. K. P. Salve Institute of Medical Sciences & Research Centre and Lata Mangeshkar Hospital, Nagpur, IND; 2 Orthopaedic Surgery, N. K. P. Salve Institute of Medical Sciences & Research Centre and Lata Mangeshkar Hospital, Nagpur, IND; 3 Orthopaedics, N. K. P. Salve Institute of Medical Sciences & Research Centre and Lata Mangeshkar Hospital, Nagpur, IND

**Keywords:** operative time, union, kujala patellofemoral score, tibia fractures, infrapatellar nailing, supraptellar nailing

## Abstract

Background and objective

Intramedullary nailing can be considered the current gold standard for the treatment of diaphyseal tibial fractures. Nailing ensures good fracture stability, protection against malalignment, and quick mobilization. The suprapatellar (SP) approach of tibial nailing in the semi-extended position has recently been recommended as a safe and effective surgical technique; it has been gaining significant attention in the orthopedic literature, with fewer complications and reoperations. The approach has been shown to facilitate a reduction in fractures around the knee joint in the semi-extended position, and the extended position of the lower leg allows for easier fluoroscopic imaging. In this study, we aimed to compare the outcomes between SP and infrapatellar (IP) approaches of intramedullary nailing in patients with extra-articular tibial fractures.

Method

A randomized control trial was conducted over a period of 1.5 years at our tertiary care hospital after obtaining approval from its institutional ethics committee. A total of 60 patients with extra-articular tibial fractures were included in the study, with 30 patients each in the SP nailing group and the IP nailing group, based on randomized sampling and with the help of radiological exposure in SP and IP nailing as per a previous study. The groups were then compared in terms of KUJALA patellofemoral knee score, operative time, radiation exposure, and time of union.

Results

When comparing both groups, those treated with the SP approach had better outcomes, including reduced radiation exposure, less pain, decreased operative time, better KUJALA patellofemoral knee score, and faster union.

Conclusion

Based on our findings of the comparison between SP and IP nailing approaches of extra-articular tibial fractures, the former leads to better and safer outcomes than the latter.

## Introduction

Tibial fractures are the most common long-bone fractures, with the highest prevalence in men between the ages of 15 and 19 years, and in women between the ages of 85 and 95 years. The average age of a patient sustaining a tibial fracture is 37 years, with an average of 31 years in men and 54 years in women. Tibial fractures are caused by direct injuries, including high-energy bending, low-energy bending, and penetrating wounds, as well as indirect injuries, including torsional mechanisms and stress fractures [1].

The tibia is often involved in open fractures due to its subcutaneous location. Severe open fractures are associated with a high rate of complications and poor long-term outcomes. In comparison to other types of fractures, tibial fractures have higher rates of malunion and non-union. Approximately 80% of tibial fractures are associated with fibula fractures [1].

The infrapatellar (IP) approach is a widely accepted treatment modality for extra-articular tibial fractures, and it is characterized by the effective management of both simple and segmental fractures of the tibia. The IP approach has several advantages, such as a shorter operative time, faster union, and early mobilization [2]. Another method, the suprapatellar (SP) approach, has recently gained wide acceptance due to its superior results compared to the IP approach in terms of fracture union time, operative time, and radiological exposure [3]. Both IP and SP approaches for interlock intramedullary tibia nailing are effective methods but each has its own complications. In this study, our objective was to compare the SP approach with the IP approach of intramedullary nailing in patients with extra-articular tibial fractures.

## Materials and methods

The present study was conducted from December 1, 2020, to October 31, 2022, at a tertiary care hospital. The study population included all patients with extra-articular tibial fractures admitted to the orthopedics ward and who fulfilled the inclusion criteria. In light of the ongoing coronavirus disease 2019 (COVID-19) pandemic, the convenient sampling technique was used, as it was considered the most suitable method for us. Patients who met the inclusion criteria were asked to participate in the study, which comprised all extra articular-tibia fractures (closed fractures and up to compound grade 2 fractures) [4] and patients aged 18 years and above. The exclusion criteria were as follows: intra-articular fractures extending in the knee or ankle joints, compound grade 3 injuries (according to Gustilo and Anderson classification) [4], ipsilateral patella fractures, any other previous fractures with implants in ipsilateral limbs, and surgically unfit patients.

The patients were divided into two groups - the SP group and the IP group - and operated on by experienced surgeons at the institution. Each patient was taken to the operation theater and placed on a table. Spinal anesthesia was given, and a high thigh tourniquet was applied. Cleaning, painting, and draping were all done by ensuring necessary aseptic precautions. Limb exsanguination was done with the use of an Esmarch bandage for one minute, and the tourniquet was inflated to a pressure of 150 mmHg above systolic pressure.

For the IP group, the anteromedial aspect of tibial tuberosity was palpated and marked, and a skin incision 3-4 cm in length was taken. The knee was taken in complete flexion, and the patella tendon was palpated. The patellar tendon was split vertically in the center. An incision was made through the patella tendon, which ensured an accurate entrance into the bone. Then, a curved awl was used to make an entry point just above and medial to the tibial tuberosity. From the entry made with the awl, a guide wire was passed with a slight bend of the tip. Upon entry into the canal, a typical grating sensation was felt. The fracture fragments were aligned manually for smooth passing of the wire. A nail, whose length was determined preoperatively, was then attached with a threaded bolt to the proximal zig and kept aside. Serial reaming of the canal was done. The nail was inserted over the guide wire and further into the medullary canal in a rotating motion by applying pressure. For the placement of the distal screw, distal locking was done under fluoroscopy guidance using a free-hand 4.5-mm bolt of appropriate length. For the placement of the proximal screw, proximal locking was done through a proximal zig using sleeves through a stab incision over the medial side. The screw length was measured with a depth gauge, and a 4.5-mm bolt was placed and checked under fluoroscopy.

**Figure 1:**
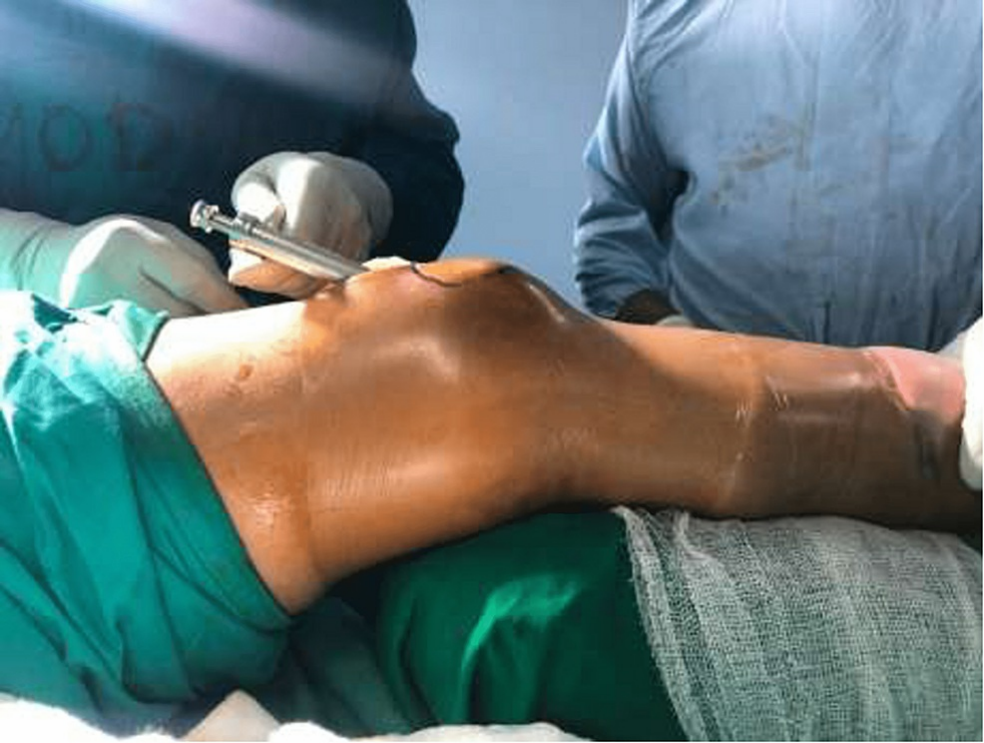
Intraoperative clinical photograph of the suprapatellar approach - image 1

**Figure 2:**
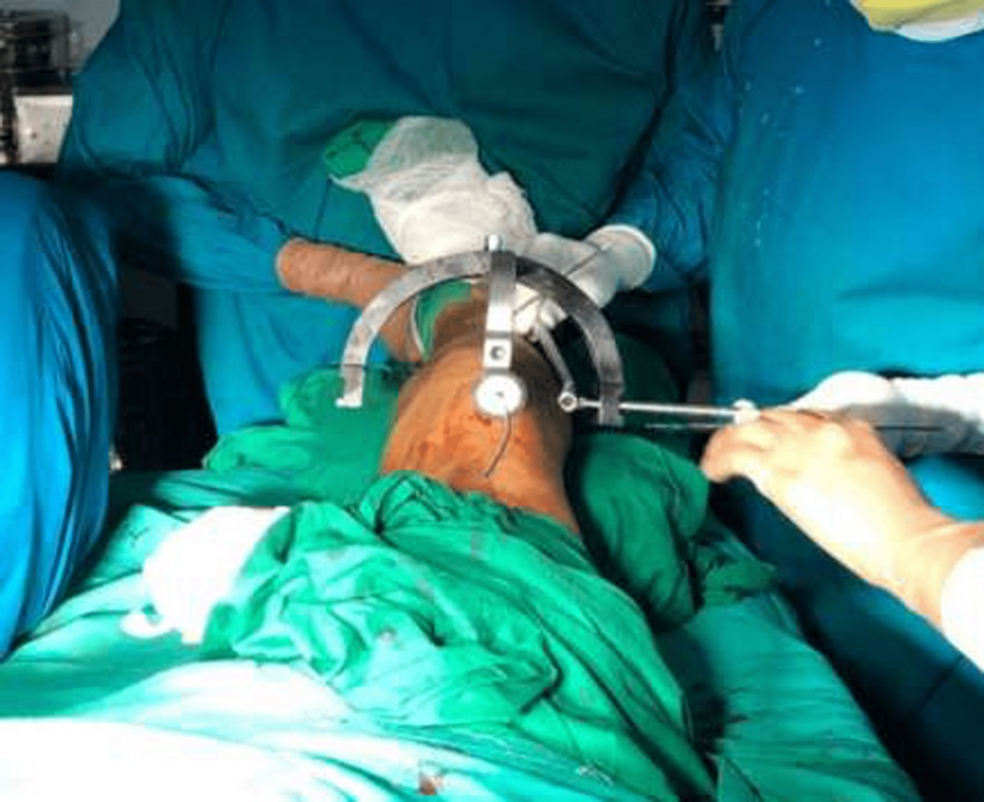
Intraoperative clinical photograph of the suprapatellar approach - image 2

For the SP group, the patient was placed in the supine position on the operating table, and the affected leg was placed with a bolster under the knee joint, thereby flexing it to 25-30 degrees. The C-arm was placed on the contralateral side. A 2- to 3-cm vertical skin incision was made above the base of the patella. The quadriceps tendon was bluntly dissected, and a vertical central split was performed in the tendon. An entry tube was then inserted, as shown in Figures 1-2.

**Figure 3:**
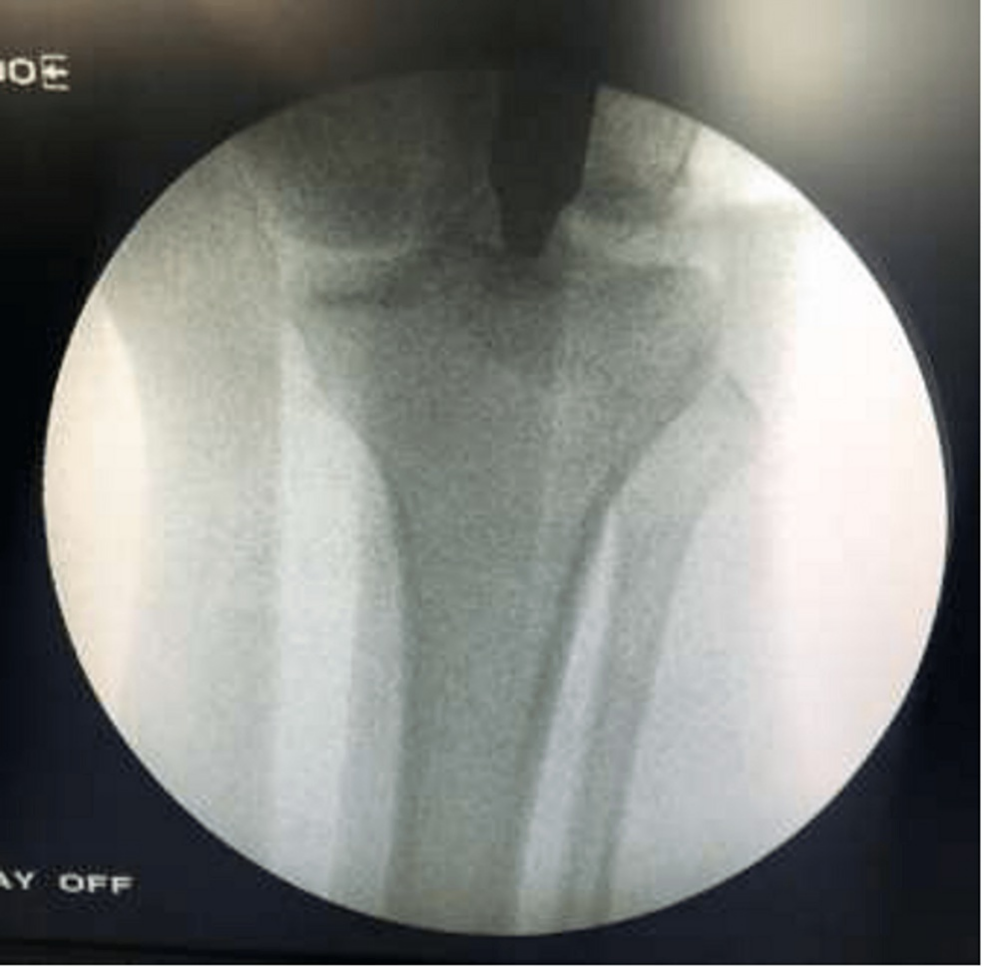
C-arm image (anteroposterior view) of the knee showing the entry point

The entry point on the anteroposterior view was located 10 mm in the lateral direction from the center of the tibial plateau and lateral to the tibial tubercle, as seen in Figure 3.

**Figure 4:**
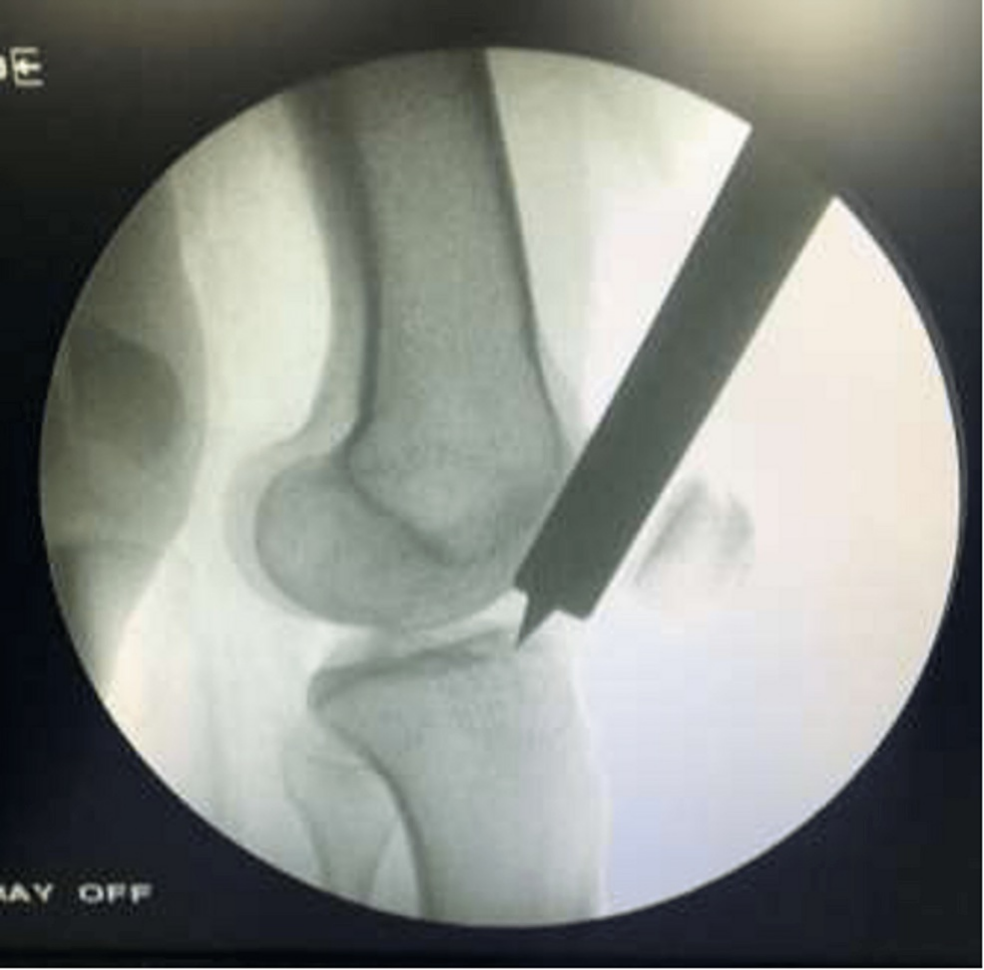
C-arm image (lateral view) of the knee showing the entry point

On the lateral view, the entry point was anterior to the anterior articular margin, as shown in Figure 4.

**Figure 5:**
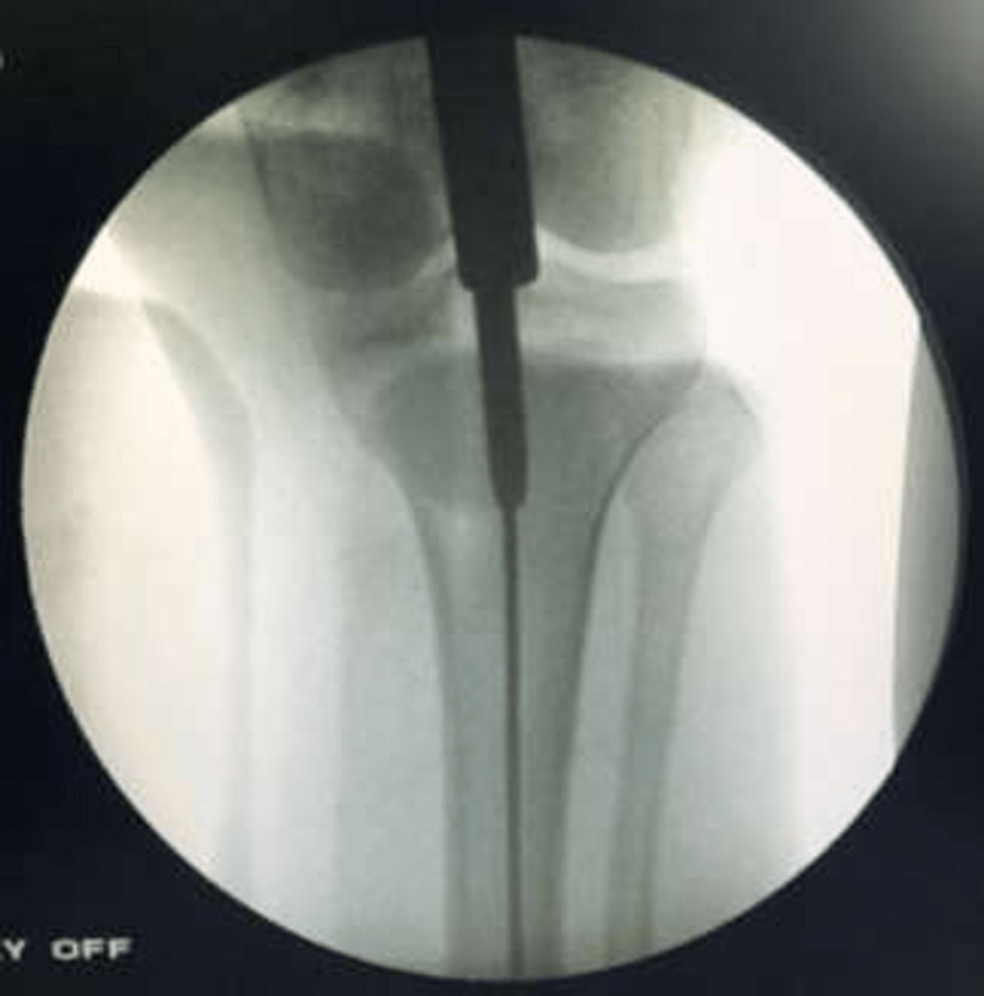
C-arm image (anteroposterior view) of the introduction of the guide wire through the sleeve

**Figure 6:**
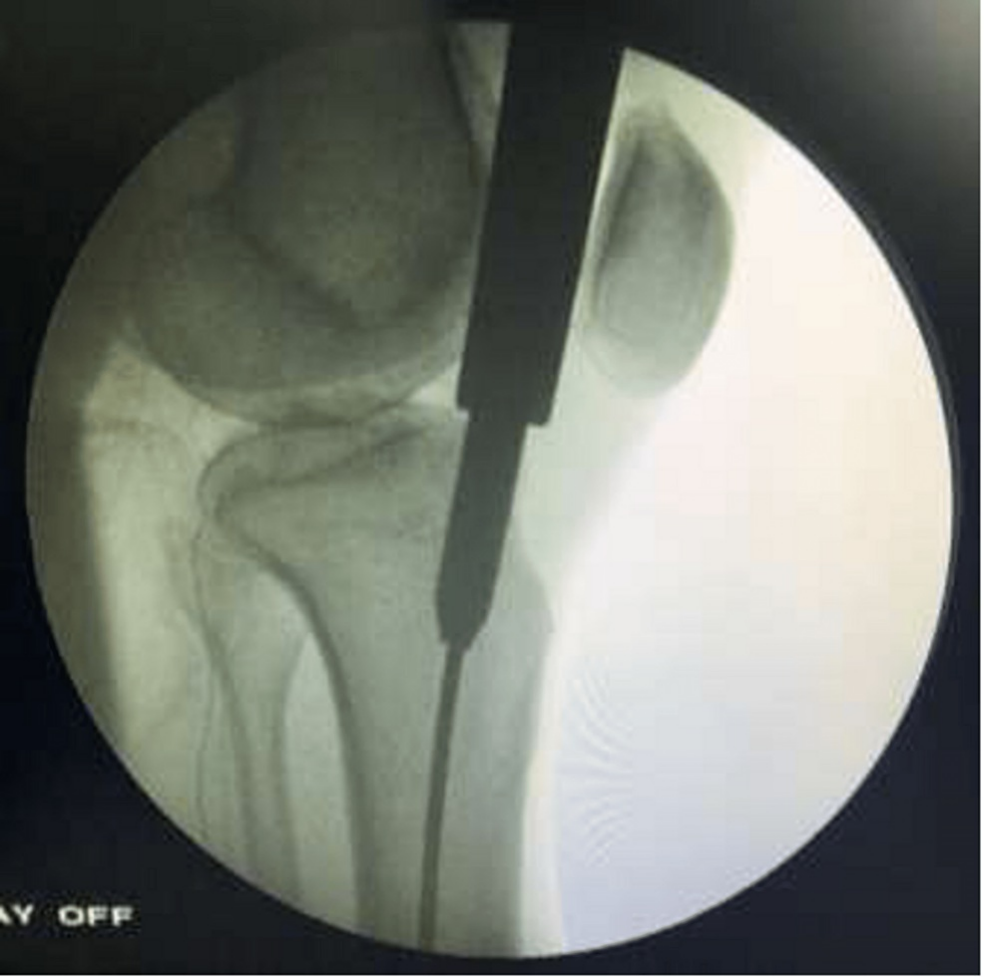
C-arm image (lateral view) of the introduction of the guide wire through the sleeve

The guide wire was then inserted into the medullary canal, as seen in Figures 5-6.

**Figure 7:**
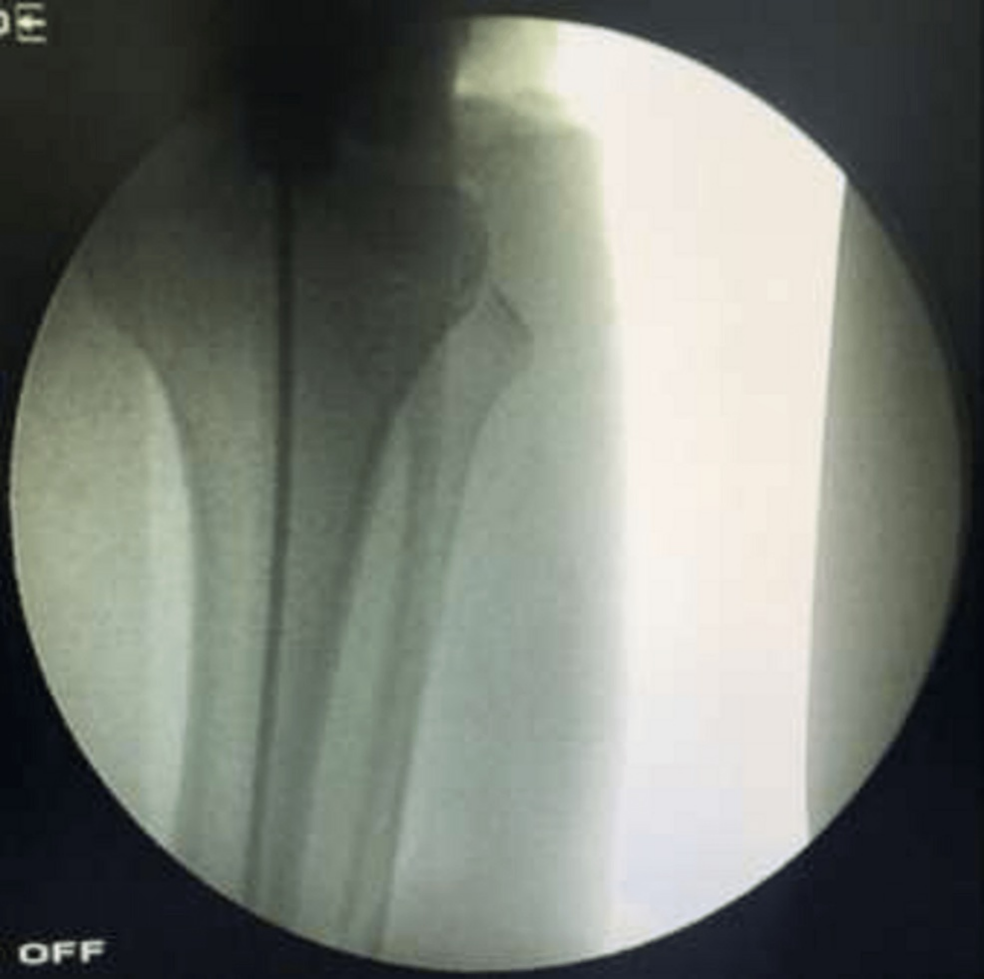
C-arm image (anteroposterior view) confirming correct guide wire placement in the tibial intramedullary canal - image 1

**Figure 8:**
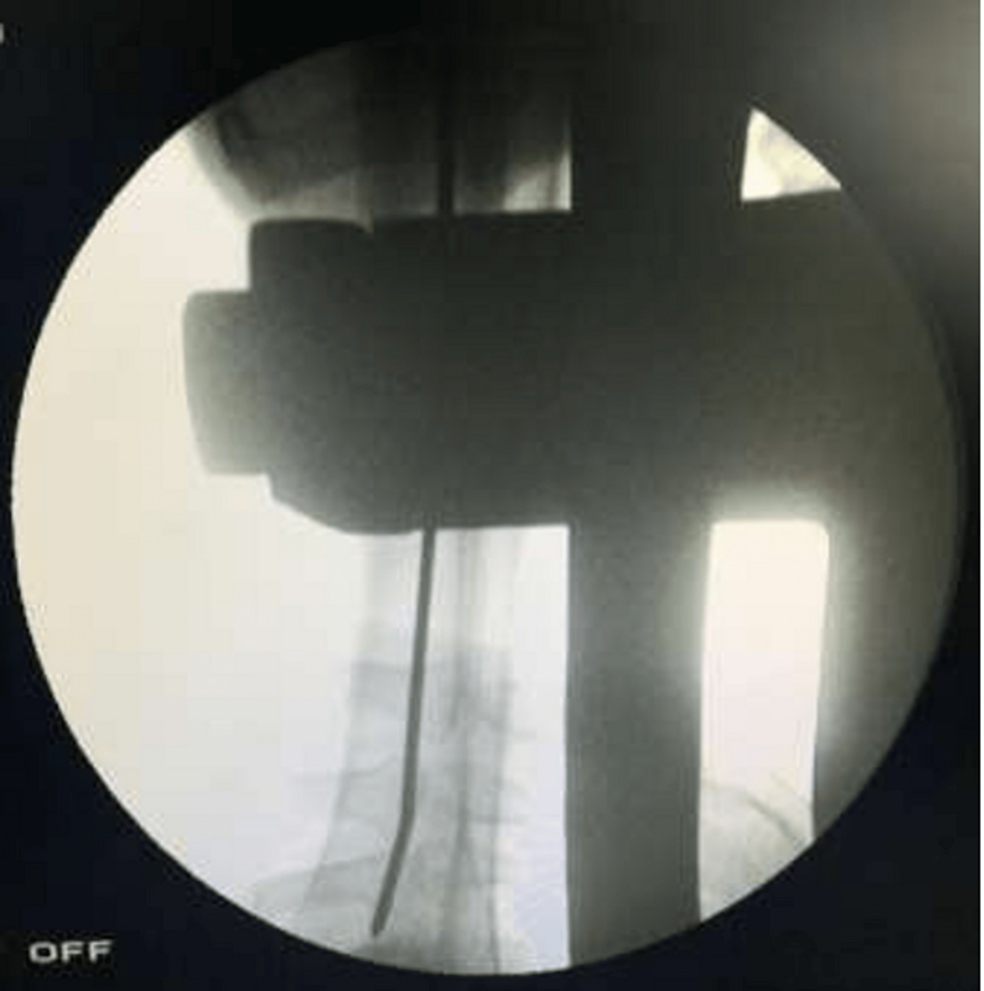
C-arm image (anteroposterior view) confirming correct guide wire placement in the tibial intramedullary canal - image 2

The protection sleeve was then inserted. Through the sleeve over the guide wire, the medullary canal was opened to a depth of 4-6 cm in the proximal tibia with a short reamer. A ball-tip guide was inserted into the medullary canal and advanced past the fracture level and down to the distal tibia. Fluoroscopic imaging was used in both planes to verify that the wire was within the medullary canal, as seen in Figures 7-8.

**Figure 9:**
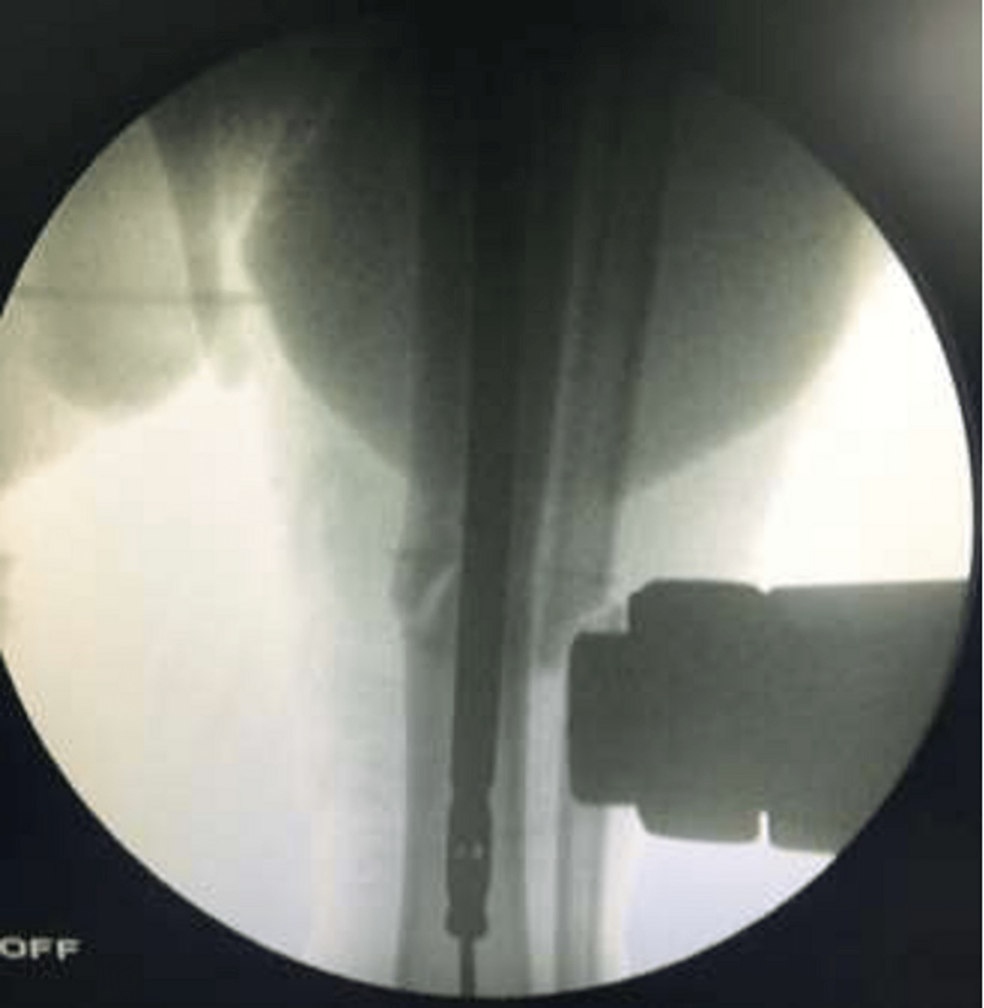
C-arm image of nail insertion over the guide wire

**Figure 10:**
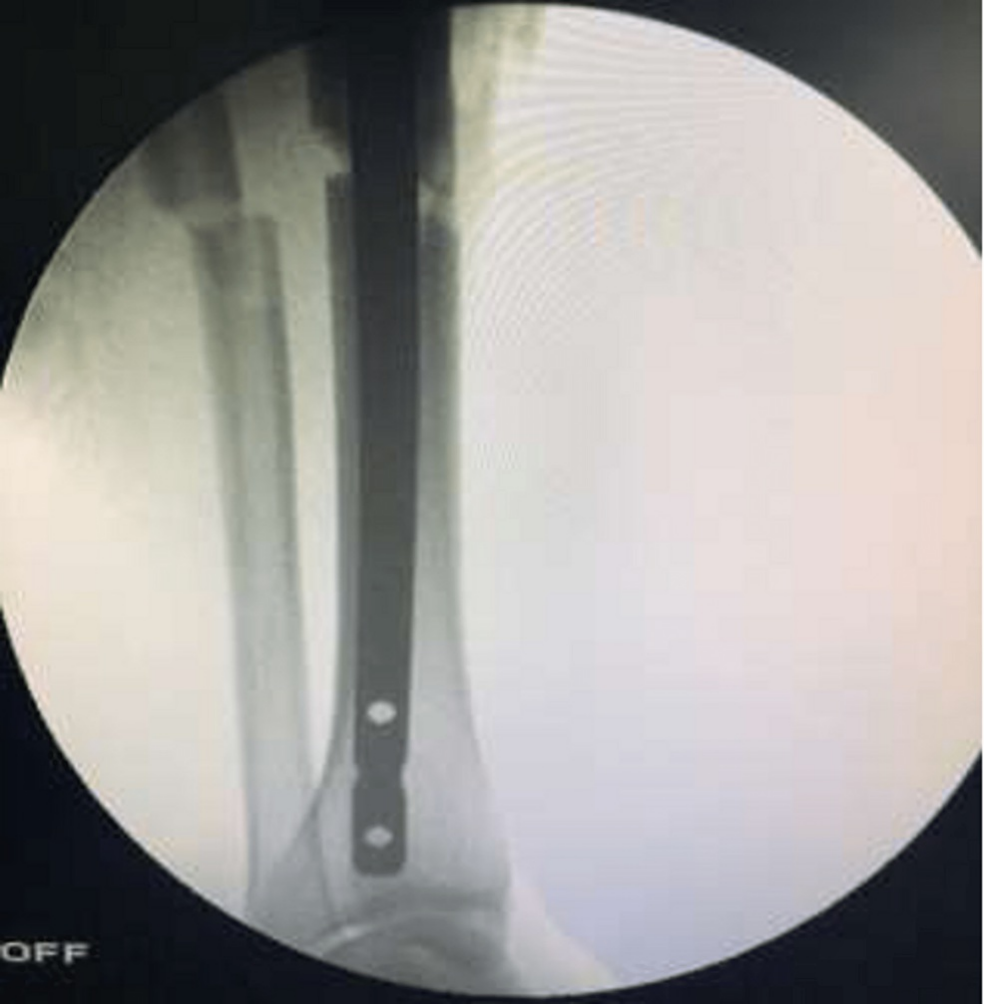
C-arm image (lateral view) of nail insertion over the guide wire

For nail insertion, the length of the nail was determined by using a proper measuring guide, as seen in Figures 9-10.

The reaming was performed through the protection sleeve. Depending on the length of the tibia, the reamer used at the SP entry was longer than that at the IP entry. Typical reaming was then done to a diameter that was 1-1.5 mm larger than that of the nail. The inner part of the sleeve protector was removed before the nail was inserted.

For the placement of the distal screws, distal locking was done under fluoroscopy guidance using a free-hand 4.5-mm bolt of appropriate length. The placement of the proximal screws was done through a proximal zig using the sleeves through a stab incision over the medial side and anteromedial side. The screw length was measured with the depth gauge, and a 4.5-mm bolt was inserted into the nail.

**Figure 11:**
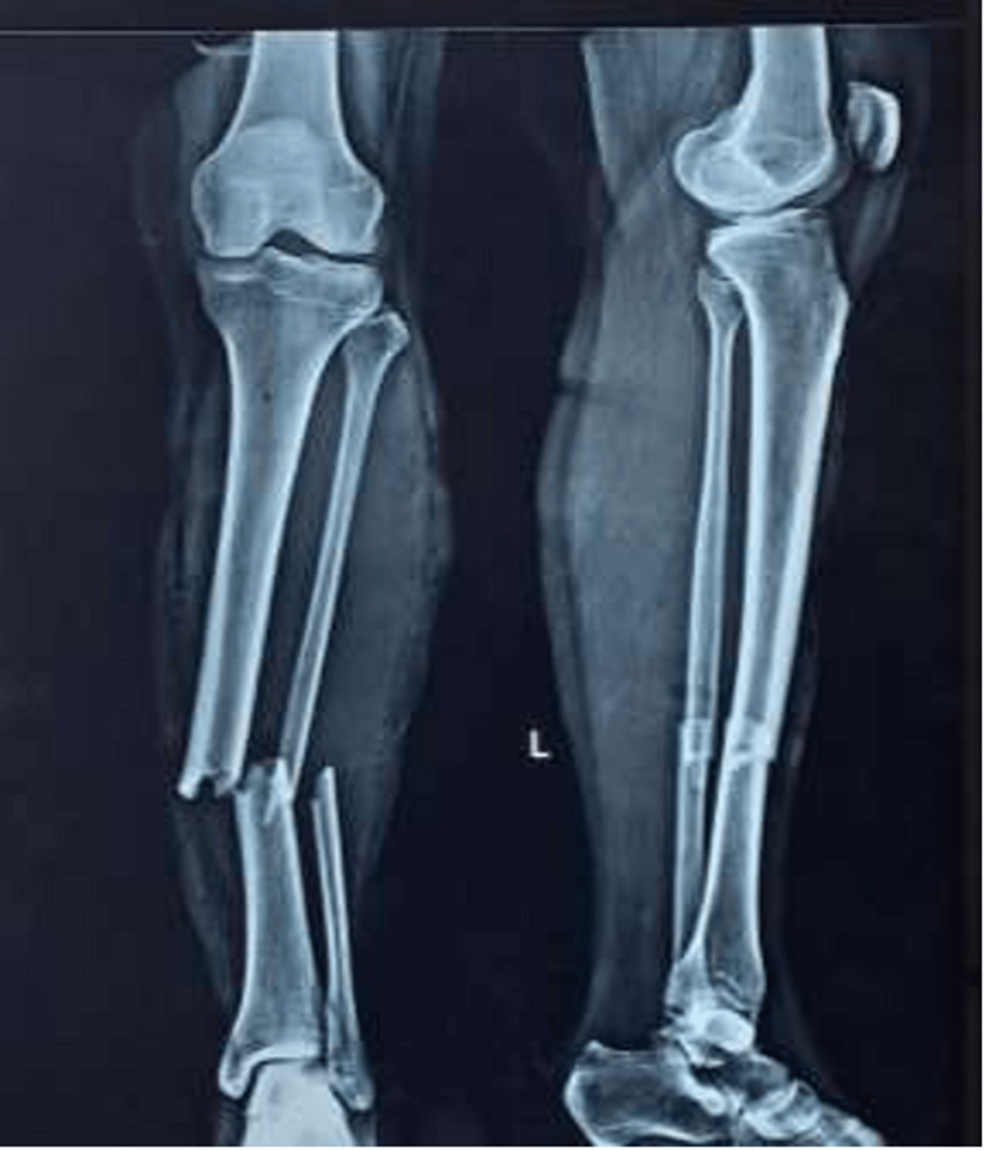
Preoperative radiograph of the leg

Fluoroscopy was used in both views to verify that the nail was not protruding into the knee. The knee joint was given a thorough saline wash to clear the joint space from any kind of debris. A preoperative radiograph was then taken (Figure 11).

**Figure 12:**
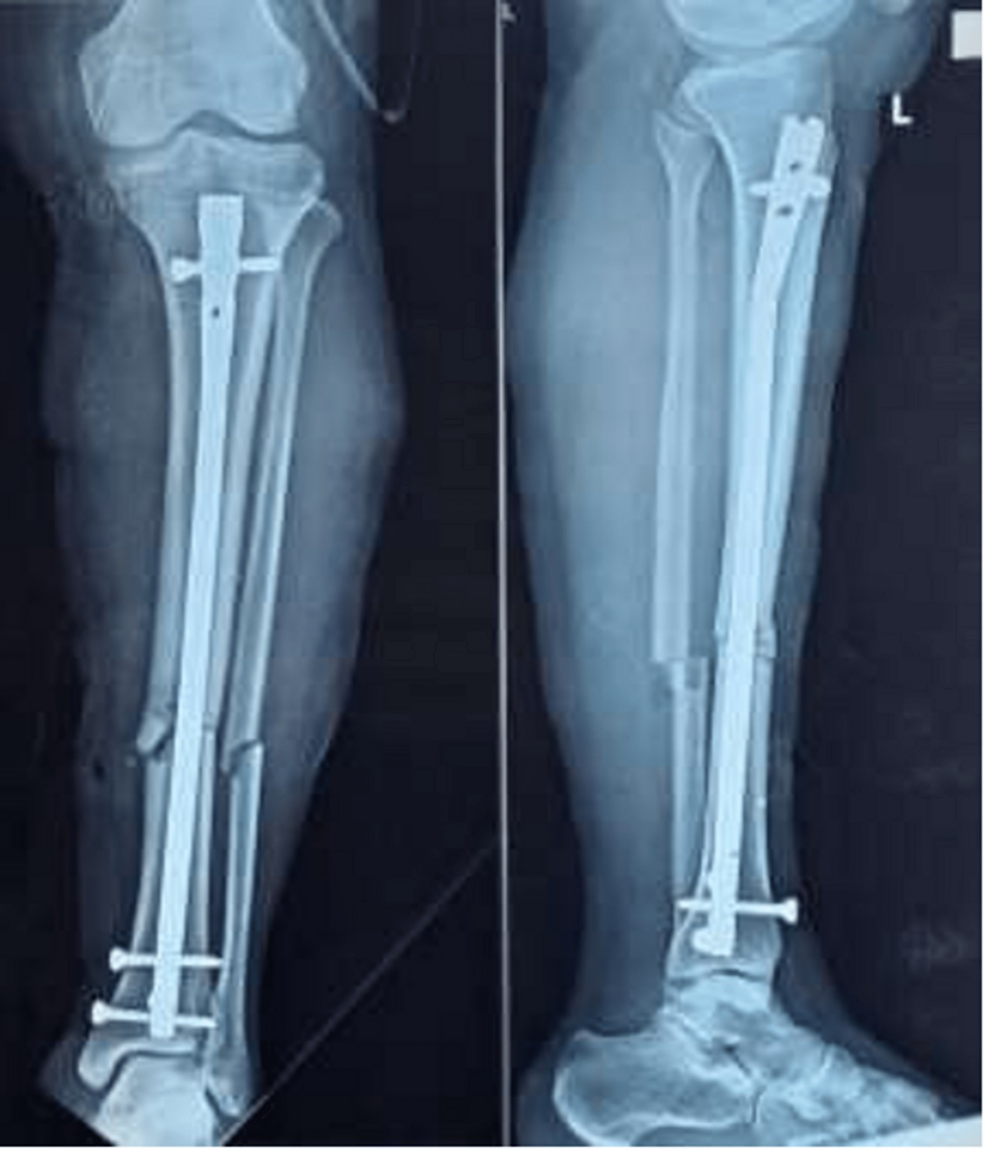
Immediate postoperative radiograph

**Figure 13:**
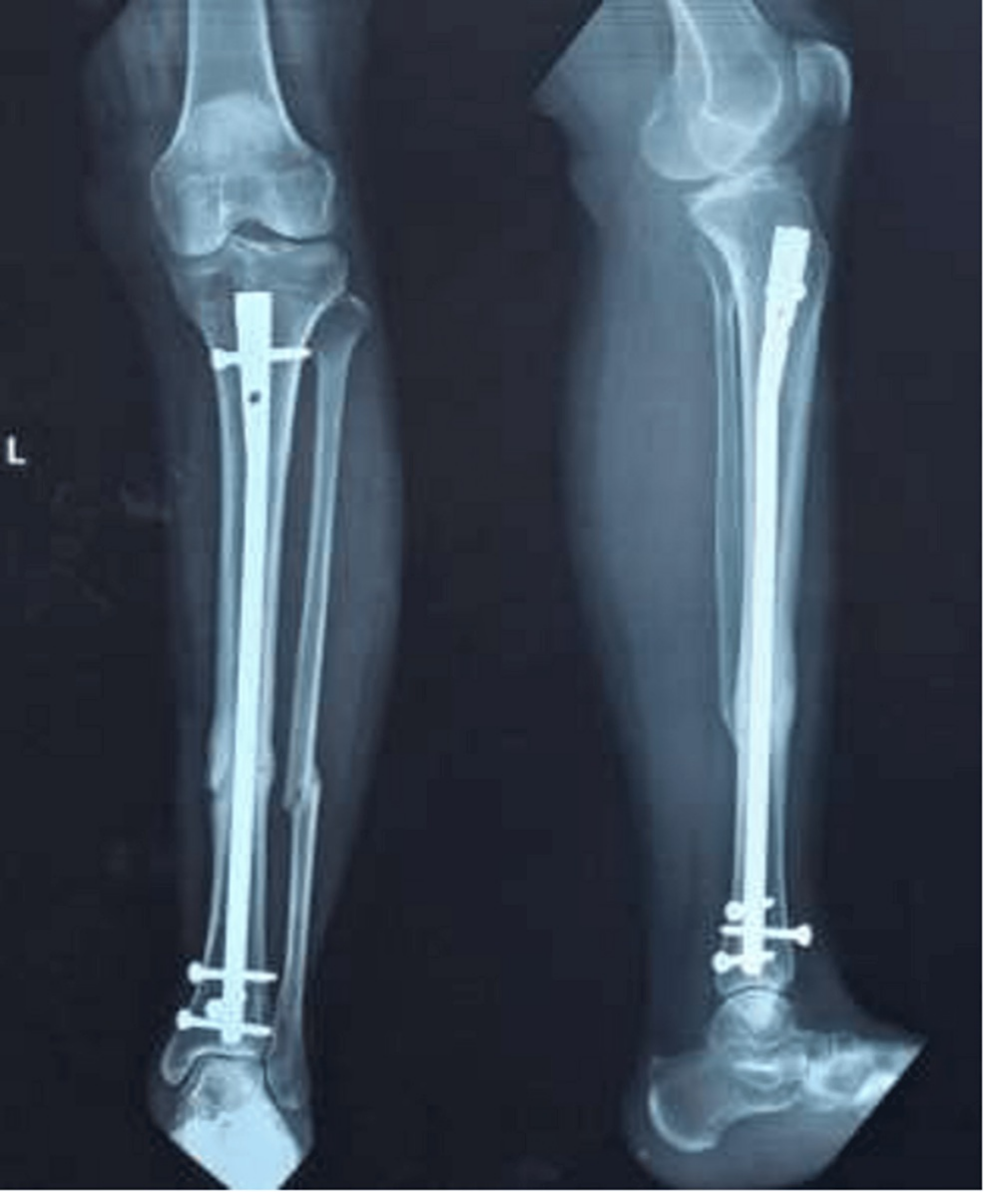
One-month follow-up radiograph

**Figure 14:**
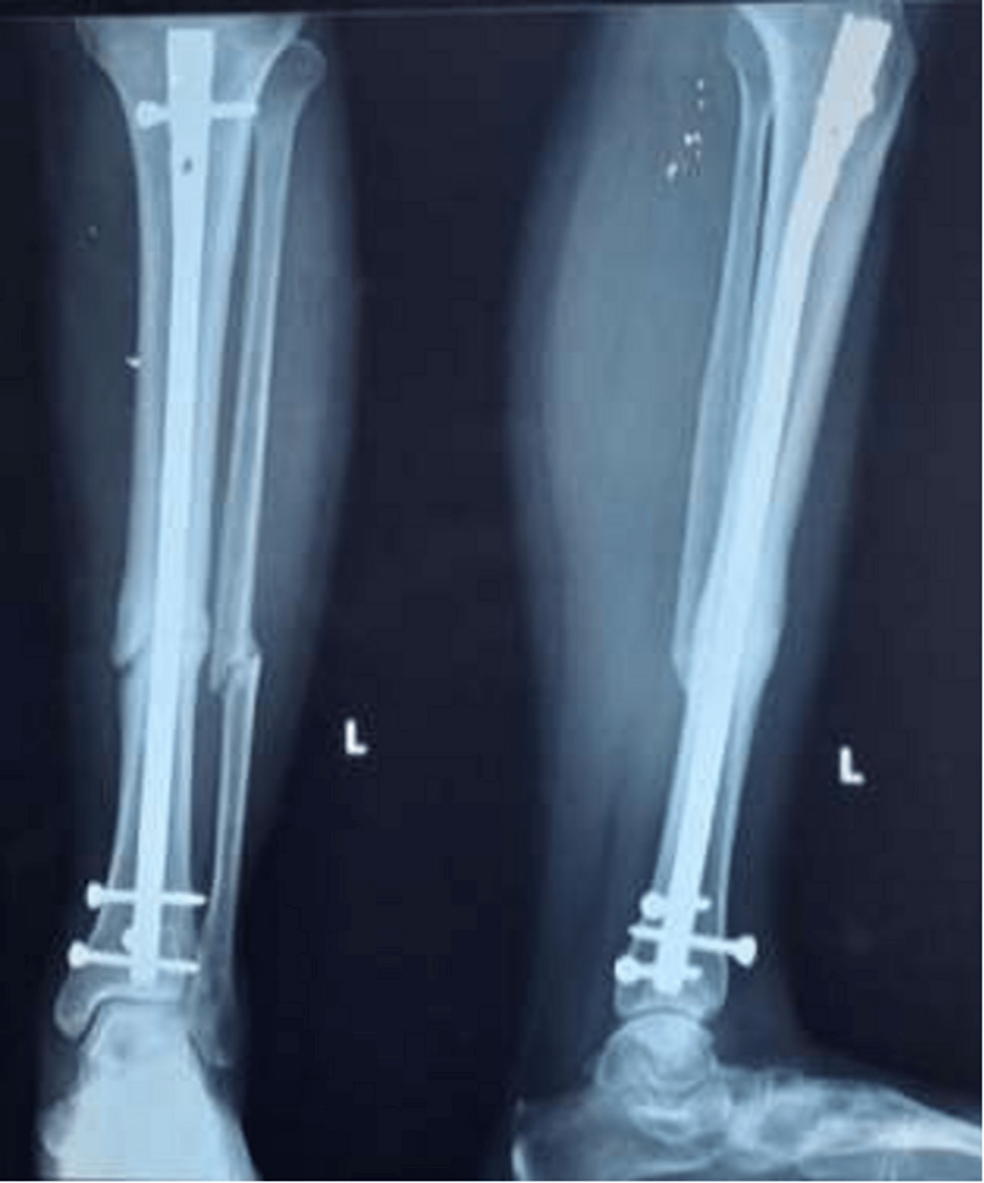
Six-month follow-up radiograph

The patients were followed up postoperatively with radiography at 0, one, and six months, as seen in Figures 12-14.

A radiological assessment was done at four weeks postoperatively, and evidence of callous formation was seen. The final outcomes were measured with respect to time of union, operative time, complications, radiological exposure, and functional outcomes assessed using the KUJALA patellofemoral score. These parameters were measured for a duration of six months, and the results were analyzed by using appropriate statistical tests.

The study was approved by the institutional review board of the N. K. P. Salve Institute of Medical Sciences & Research Centre and Lata Mangeshkar Hospital, Digdoh Hills, Hingna Road, Nagpur (approval no: 60/2021).

## Results

Table 1 presents a comparison of the mean postoperative KUJALA scores between the groups. An unpaired Student’s t-test showed a significant difference between the groups (p=0.0001), with a higher mean postoperative KUJALA score in the SP group.

**Table 1:** Comparison of immediate postoperative mean KUJALA scores between the groups *Statistically significant SD: standard deviation

Immediate postop KUJALA score	Infrapatellar group, mean ± SD	Suprapatellar group, mean ± SD	P-value (unpaired t-test)
	32.76 ± 1.52	35.00 ± 2.85	0.001*
Range	31-34	29-39	

Table 2 provides a comparison of the mean postoperative KUJALA scores at one month between the groups. An unpaired Student’s t-test showed a significant difference between the groups (p=0.0001), with a higher score in the SP group at one month.

**Table 2:** Comparison of mean postoperative KUJALA scores between the groups at 1 month *Statistically significant SD: standard deviation

Postop KUJALA score at 1 month	Infrapatellar group, mean ± SD	Suprapatellar group, mean ± SD	P-value (unpaired t-test)
	42.53 ± 1.27	45.03 ± 2.74	0.0001*
Range	41-45	40-49	

Table 3 shows a comparison of mean postoperative KUJALA scores at six months. An unpaired Student’s t-test showed a significant difference between the groups (p=0.0001), with a higher score in the SP group at six months.

**Table 3:** Comparison of mean postoperative KUJALA scores between the groups at 6 months *Statistically significant SD: standard deviation

Postop KUJALA score at 6 months	Infrapatellar group, mean ± SD	Suprapatellar group, mean ± SD	P-value (unpaired t-test)
	59.40 ± 4.51	72.90 ± 3.40	0.0001*
Range	51-68	65-79	

Table 4 shows a comparison of mean radiological exposure (in cGy/cm^2^) between the groups. An unpaired Student’s t­­-test showed a highly statistically significant difference between the groups (p=0.0001), with higher radiological exposure in the IP group.

**Table 4:** Comparison of mean radiological exposure between the groups *Statistically significant SD: standard deviation

Radiological exposure (cGy/cm^2^)	Infrapatellar group, mean ± SD	Suprapatellar group, mean ± SD	P-value (unpaired t-test)
	62.87 ± 5.56	40.73 ± 3.88	0.0001*
Range	52-72	32-49	

Table 5 provides a comparison of mean operative time (minutes) between the groups. An unpaired Student’s t-test showed a highly statistically significant difference between the groups (p=0.0001), with a higher mean operative time in the IP group.

**Table 5:** Comparison of mean operative time between the groups *Statistically significant SD: standard deviation

Operative time (minutes)	Infrapatellar group, mean ± SD	Suprapatellar group, mean ± SD	P-value (unpaired t-test)
	147.33 ± 8.96	110.20 ± 5.79	0.0001*
Range	127-160	101-119	

Table 6 provides a comparison of the mean time of union of fractures (months) between the groups. An unpaired t-test showed a significant difference between the groups (p=0.0001), with a longer mean union time in the IP group.

**Table 6:** Comparison of the mean time of union of fractures between the groups *Statistically significant SD: standard deviation

Union time (months)	Infrapatellar group, mean ± SD	Suprapatellar group, mean ± SD	P-value (unpaired t-test)
	7.00 ± 1.14	5.60 ± 1.10	0.001*
Range	5-9	4-8	

## Discussion

Tibial shaft fractures account for 3% of all fractures in adults, and the conventional treatment for the condition involves intramedullary interlock nailing. Until recently, the IP approach was the most commonly used method of nailing, but the SP approach, with the knee in a semi-extended position, has gained popularity because of its many advantages, such as easy reduction, accurate entry, short operative time, and low exposure to fluoroscopic radiation [5].

There have been concerns of malalignment with regard to the IP approach, with issues including knee hyperflexion, and difficulty in achieving and holding reduction in upper-third and lower-third fractures in hyperflexion. Various studies have reported a significantly lower incidence of malalignment when using the SP approach. While it is usually employed in proximal tibia fractures, better outcomes have also been seen in lower-third fractures. The SP approach does not require hyperflexion and repeated manipulation for positioning, which are prerequisites in IP nailing. Thus, the leg remains in a semi-extended position during the entire operative duration [5].

The present study was conducted at our tertiary care institute with a view to comparing the functional outcomes of intramedullary interlock nailing between SP and IP approaches in patients with extra-articular tibial fractures. We included a total of 60 cases in our study, of which 30 were in the IP group, and 30 were in the SP group.

In our study, the Student’s t-test showed a significant difference between the mean duration for the union of fracture in both groups (p=0.0001), with a longer mean union time in the IP group. Metcalf et al. [6] have observed that there was a lower risk of malunion with SP nailing (20% in IP vs. 4% in SP nails), whereas Ponugoti et al. [7], in their systematic review, found that the difference between both approaches was not significant in terms of malunion. Cicekli et al. [8] found that the mean union time was 13 weeks in SP patients and 16 weeks in IP patients, which is comparable to our findings. However, Lu et al. [9] observed no statistically significant difference in union time between the two patient types (p=0.550).

In our study, the mean operative time was 147.33 ± 8.96 minutes in the IP group and 110.20 ± 5.79 minutes in the SP group. Student’s unpaired t-test showed a highly statistically significant difference between the groups (p=0.0001), with a longer operative time in the IP group. Similar findings were seen in a study by Al-Azzawi et al. [5], where the mean operative time was 140 ± 26.5 minutes for the IP group and 112 ± 36.2 minutes for the SP group (p<0.05). Cicekli et al. [8] also had similar findings, with a shorter average operative time in the SP group. Gao et al. [10] reported mean operative times of 67.91 ± 7.38 minutes and 85.32 ± 9.17 minutes in the SP and IP groups, respectively, which is similar to our findings. Lu et al. [9] observed a longer mean operative time in the SP group than in the IP group, whereas MacDonald et al. [11] found no difference in operating time between the groups. In their meta-analysis, Ponugoti et al. [7] found that the mean operative time was not significantly different between the SP and IP groups.

In the present study, Student’s unpaired t-test showed a highly statistically significant difference in mean radiological exposure between the groups (p=0.0001), with higher radiological exposure in the IP group. This is in line with the findings of Al-Azzawi et al. [5], where the mean radiation was 78.33 ± 35.4 cGY/cm^2^ in the IP group and 38 ± 30.6 cGY/cm^2^ in the SP group (p<0.05). In contrast, studies by Cicekli et al. [8] and Jones et al. [12] did not find significant differences in radiation dose between the IP and SP groups.

Although an overall improvement in the KUJALA score was seen in both the IP and SP groups, the SP group saw much greater improvement. Student’s unpaired t-test showed a significant difference in postoperative KUJALA scores at one month and six months between the groups (p=0.0001), with higher scores in the SP group. Similar findings were seen in a study by Jones et al. [12], with higher KUJALA scores in the SP group than in the IP group. However, a similar study by Ozcan et al. [13] on transpatellar, medial parapatellar, and suprapatellar nailing did not find any statistical difference in KUJALA scores between the three groups. Cicekli et al. [8] observed KUJALA scores of 87.82 and 83.37 in SP and IP groups, respectively - a difference that was not statistically significant (p=0.098) - which was in contrast with the findings of our study. Overall, we observed that functional outcomes improved post-surgery in our cases.

Eastman et al. [14] reported good alignment in upper-third fractures via the SP approach. They concluded that angular forces could be overcome by the semi-extended position of the SP approach. Zelle et al. [15] reported better alignment of fractures by using the SP approach. Franke et al. [16] reported that the entry point used in the SP approach was more in line with the anatomical axis of the tibia. Sanders et al. [17] reported only one improper alignment in 39 fractures where SP nailing was done. We found the SP approach to be better in terms of functional outcomes, operative time, radiation exposure, and union time.

Limitations of the study

This study has one limitation that needs to be taken into account: abrasions or wounds over the SP area that made it difficult to make an incision for SP nailing.

## Conclusions

Based on our findings in comparing the outcomes between SP and IP nailing of extra-articular tibial fractures, we found that the former was a better and safer method than the latter.

## References

[1] (2007). Handbook of Fractures: Third edition. https://books.google.co.in/books/about/Handbook_of_Fractures.html?id=1x6ZQgAACAAJ&redir_esc=y.

[2] Hooper GJ, Keddell RG, Penny ID (1991). Conservative management or closed nailing for tibial shaft fractures. A randomised prospective trial. J Bone Joint Surg Br.

[3] Wang C, Chen E, Ye C, Pan Z (2018). Suprapatellar versus infrapatellar approach for tibia intramedullary nailing: a meta-analysis. Int J Surg.

[4] Ganesan GR, Rajappa S (2014). The mess: is it must?. Int J Clin Trials.

[5] Al-Azzawi M, Davenport D, Shah Z, Khakha R, Afsharpad A (2021). Suprapatellar versus infrapatellar nailing for tibial shaft fractures: a comparison of surgical and clinical outcomes between two approaches. J Clin Orthop Trauma.

[6] Metcalf KB, Du JY, Lapite IO (2021). Comparison of infrapatellar and suprapatellar approaches for intramedullary nail fixation of tibia fractures. J Orthop Trauma.

[7] Ponugoti N, Rudran B, Selim A, Nahas S, Magill H (2021). Infrapatellar versus suprapatellar approach for intramedullary nailing of the tibia: a systematic review and meta-analysis. J Orthop Surg Res.

[8] Cicekli O, Topçu HN, Kochai A, Sukur E, Turker M (2019). Comparison of suprapatellar and infrapatellar tibial nailing: more anatomic entry point and fracture reduction via the suprapatellar approach. Int J Clin Exp Med.

[9] Lu Y, Wang G, Hu B (2020). Comparison of suprapatellar versus infrapatellar approaches of intramedullary nailing for distal tibia fractures. J Orthop Surg Res.

[10] Gao Z, Han W, Jia H (2018). Suprapatellar versus infrapatellar intramedullary nailing for tibal shaft fractures: a meta-analysis of randomized controlled trials. Medicine (Baltimore).

[11] MacDonald DR, Caba-Doussoux P, Carnegie CA, Escriba I, Forward DP, Graf M, Johnstone AJ (2019). Tibial nailing using a suprapatellar rather than an infrapatellar approach significantly reduces anterior knee pain postoperatively: a multicentre clinical trial. Bone Joint J.

[12] Jones M, Parry M, Whitehouse M, Mitchell S (2014). Radiologic outcome and patient-reported function after intramedullary nailing: a comparison of the retropatellar and infrapatellar approach. J Orthop Trauma.

[13] Ozcan C, Turkmen I, Sokucu S (2020). Comparison of three different approaches for anterior knee pain after tibia intramedullary nailing. Eur J Trauma Emerg Surg.

[14] Eastman J, Tseng S, Lo E, Li CS, Yoo B, Lee M (2010). Retropatellar technique for intramedullary nailing of proximal tibia fractures: a cadaveric assessment. J Orthop Trauma.

[15] Zelle BA, Boni G, Hak DJ, Stahel PF (2015). Advances in intramedullary nailing: suprapatellar nailing of tibial shaft fractures in the semiextended position. Orthopedics.

[16] Franke J, Hohendorff B, Alt V, Thormann U, Schnettler R (2016). Suprapatellar nailing of tibial fractures-Indications and technique. Injury.

[17] Sanders RW, DiPasquale TG, Jordan CJ, Arrington JA, Sagi HC (2014). Semiextended intramedullary nailing of the tibia using a suprapatellar approach: radiographic results and clinical outcomes at a minimum of 12 months follow-up. J Orthop Trauma.

